# Choroidal Vascularity Map in Unilateral Central Serous Chorioretinopathy: A Comparison with Fellow and Healthy Eyes

**DOI:** 10.3390/diagnostics11050861

**Published:** 2021-05-11

**Authors:** Niroj Kumar Sahoo, Sumit Randhir Singh, Oliver Beale, Gideon Nkrumah, Mohammed Abdul Rasheed, Asiya Jabeen, Kiran Kumar Vupparaboina, Mohammed Nasar Ibrahim, Filippo Tatti, Khushboo Chandra, Michele Lanza, Claudio Iovino, Enrico Peiretti, Jay Chhablani

**Affiliations:** 1 Department of Retina and Vitreous, L V Prasad Eye Institute, Vijayawada 521134, India; nirojsahoo71@gmail.com; 2 Jacobs Retina Center, Shiley Eye Institute, University of California San Diego, San Diego, CA 92697, USA; sumit.jipmer@gmail.com; 3 UPMC Eye Centre, University of Pittsburgh, Pittsburgh, PA 15213, USA; oliver.m.beale@gmail.com (O.B.); blayd147@gmail.com (G.N.); Kiran1559@gmail.com (K.K.V.); 4 School of Optometry and Vision Science, University of Waterloo, Waterloo, ON N2L 3G1, Canada; hcu.sahil@gmail.com (M.A.R.); asiyajabeen044@gmail.com (A.J.); 5 Department of Electrical Engineering, Indian Institute of Technology, Hyderabad 502285, India; EE12P1004@iith.ac.in; 6 Department of Surgical Sciences, Eye Clinic, University of Cagliari, 9121 Cagliari, Italy; filippotatti@gmail.com (F.T.); enripei@hotmail.com (E.P.); 7 Department of Vitreoretina, Disha Eye Hospitals Pvt Ltd., 88(63A) Ghosh Para Road, Barrackpore, Kolkata 700120, India; khushboo.chandra01@gmail.com; 8 Eye Clinic, Multidisciplinary Department of Medical, Surgical and Dental Sciences, University of Campania Luigi Vanvitelli, 81100 Naples, Italy; mic.lanza@gmail.com (M.L.); claudioiovino88@gmail.com (C.I.)

**Keywords:** central serous chorioretinopathy, CSCR, choroidal vascularity index, CVI, mapping, ETDRS grid

## Abstract

Background: To map the choroidal vascularity index and compare two eyes in patients with unilateral central serous chorioretinopathy (CSCR). Methods: This was a retrospective, observational study performed in patients with unilateral CSCR. Choroidal thickness (CT) and Choroidal vascularity index (CVI) were measured and mapped in various zones according to the early treatment diabetic retinopathy (ETDRS) grid. Results: A total of 20 CSCR patients (20 study and 20 fellow eyes) were included in the study. Outer nasal region CT was seen to be significantly lower than central CT (*p* = 0.042) and inner nasal CT (*p* = 0.007); outer ring CT was significantly less than central (*p* = 0.04) and inner ring (*p* = 0.01) CT in CSCR eyes. On potting all the CVI values against the corresponding CT values, a positive correlation was seen in CSCR eyes (*r* = 0.54, *p* < 0.01), which was slightly weaker in fellow eyes (*r* = 0.3, *p* < 0.01) and a negative correlation was seen in healthy eyes (*r* = −0.262, *p* < 0.01). Conclusions: Correlation between CVI and CT was altered in CSCR eyes as compared to fellow and normal eyes with increasing CVI towards the center of the macula and superiorly in CSCR eyes.

## 1. Introduction

The choroid plays a crucial role in the pathogenesis of central serous chorioretinopathy (CSCR) [1]. Being a part of the pachychoroid spectrum of diseases, it is characterized by a series of structural and functional changes in the choroidal architecture [2]. Various investigational modalities have been used to understand the biomechanics of the disease. By far, the most popular ones have been the fundus fluorescein angiogram (FFA), indocyanine green angiogram (ICG) and optical coherence tomography (OCT). Traditionally, ICG had been the only investigative modality capable of visualizing the choroid and its details [3]. Newer generation OCT machines have revolutionized the way we look at the choroid. By altering the area of segmentation in enface OCTs and OCT angiographies (OCTA), information can be obtained comparable or superior to conventional ICG without the concern of any invasive procedure [4,5,6]. The uniqueness of these modalities lies in the ease of assessment of various quantifiable parameters such as thickness, volume and indices, which were limited in traditional dye angiograms.

The concept of choroidal vascularity index (CVI) is an appealing stride in the understanding of choroidal vascular changes. It is a ratio of choroidal vessel luminal area to the total choroidal area [7]. However, the originally described method is composed of analysis of only a single cross-sectional OCT scan [7]. This provides limited information on the generalized choroidal vascular changes. This shortcoming has been circumvented in one of our previous report by utilizing a volumetric scan to quantify CVI in various zones of macula [8]. We showed that in healthy eyes, the CVI values did not change significantly across various zones. Furthermore, they did not differ significantly from the fellow eyes. However, choroid in CSCR behaves differently than normal eyes. Increase in choroidal thickness (CT) and CVI has been noted in acute and chronic CSCR eyes [9]. These changes can be generalized or local, in the form of change in choroidal vascular caliber [10]. Interestingly, even the apparently healthy looking fellow eyes of unilateral CSCR cases demonstrate abnormally higher choroidal thickness [9], and variable degrees of RPE abnormalities on multimodal imaging [2]. However, there is still no literature assessing if these choroidal changes in fellow eyes are focal or diffuse, in comparison to diseased eyes. Therefore, knowledge of the sectoral comparison for changes in choroidal parameters could add to the understanding of the disease. In this study we tried to analyze this pattern of choroidal thickness (CT) and CVI at different zones of the posterior pole in unilateral CSCR eyes and their fellow eyes.

## 2. Materials and Methods

This was a retrospective, observational study conducted at a tertiary health care centers (Italy and USA). It adhered to the tenets of declaration of Helsinki and was approved by the institutional review board. Written consent was taken from all patients before enrolling in the study. The inclusion criteria were patients above the age of 18 with no history of ocular surgery or systemic co-morbidity, having a diagnosis of unilateral acute or chronic CSCR. We excluded patients with high myopia, optic disc pathology and retinal degeneration or dystrophies that could affect the choroidal vascularity. Twenty age and sex-matched healthy eyes were also taken as a control.

### OCT Parameters

**Figure 1:**
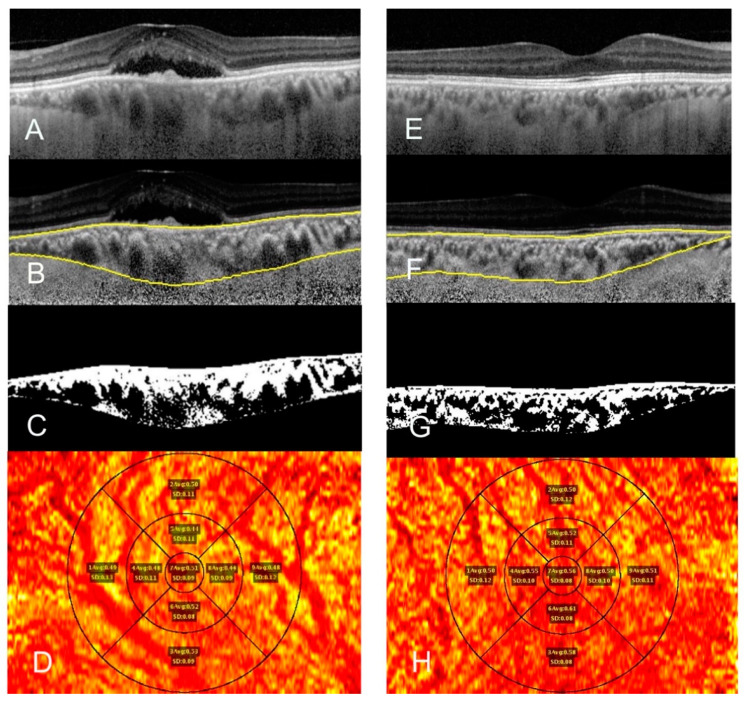
Comparison of choroidal vascularity index mapping of a 52-year-old female showing optical coherence tomography raw image of central serous chorioretinopathy (CSCR) eye (**A**) and fellow eye (**E**), choroid segmentation using an automated algorithm after shadow compensation of CSCR (**B**) and fellow eye (**F**); and binarized image (**C**,**G**). ETDRS grid-based choroidal vascular index (CVI) map images shows mean and standard deviation of CVI at the center, the 3-mm zone, and the 6-mm zone in each quadrant of CSCR eye (**D**) and fellow eye (**H**). (**D**,**H**) Red color shows choroidal vessels and yellow color shows choroidal stroma.

All patients underwent high definition enhanced depth imaging OCT scans using Spectralis OCT (Heidelberg Engineering, Heidelberg, Germany) in both eyes. Poor quality OCT scans were discarded. Raster scans were performed to cover a 20 × 20° (6 × 6 mm) field. Choroidal thickness (CT) and CVI mapping was done using a previously described method [8]. It involved shadow compensation, choroid localization, binarizing choroidal layers, three-dimensional (3D) mapping, and early treatment of diabetic retinopathy study (ETDRS) grid-based quantification [8]. CT and CVI data were isolated from each region (central, inner nasal, inner temporal, inner superior, inner inferior, outer nasal, outer temporal, outer superior, outer inferior); each quadrant (superior, inferior, nasal, temporal); and each circle (inner circle, outer circle) of the ETDRS grid [8]. The central ring had a diameter of 1 mm, centered around fovea, with rings extending 1 to 3 and 3 to 6 mm from the fovea constituted the inner and outer ring, respectively. The values were compared with each other per eye and with the fellow eye. An example of CVI mapping has been shown in Figure 1.

Statistical analysis was performed using SPSS statistical software version 20 (SPSS, Inc., Chicago, IL, USA). Values were reported as mean ± standard deviation (SD). The comparisons of CVI in different circles, subfields, and quadrants were done using repeated measures analysis of variance (ANOVA). A paired *t*-test was used to assess the difference between the two eyes. A *p*-value of <0.05 was taken as statistically significant. Surface graphs were created using Excel (Microsoft, Fremont, CA, USA) and missing mesh values were imputed using XYZ Mesh (Gray Technical). With a level of significance of 5% and power of 80%, using the subfoveal CT values for fellow eye group as 360 ± 58, for CSCR group as 429 ± 74.18 µ and attrition of 20%, the minimum sample size was found to be 18 in each group (rounded up to 20 in each group in the study) [11].

## 3. Results

A total of 20 unilateral CSCR patients (20 study and 20 fellow eyes) were included in the study. The mean age of the cohort was 48 ± 11.1 years (15 males and 5 females). Thirteen eyes had chronic CSCR and seven had acute CSCR. The fellow eyes in all the patients did not show any sub-retinal fluid or RPE changes on FFA. The mean central choroidal thickness (central 1 mm) of the CSCR eyes was 505.3 ± 124.2 µ while that of their fellow eyes was 452.8 ± 127.5 µ (*p* = 0.02). In the CSCR eyes, the overall average choroidal volume was 1.33 ± 0.70 mm^3^, with a luminal volume of 0.69 ± 0.36 mm^3^ and an overall CVI of 0.533 ± 0.038. While in the fellow eyes, the average choroidal volume was 1.19 ± 0.61 mm^3^, choroidal luminal volume was 0.62 ± 0.31 mm^3^ and the overall CVI of 0.528 ± 0.032.

### 3.1. Within Group Comparison

#### 3.1.1. CSCR Eyes

On repeated measures ANOVA, the overall change (within subject contrasts) in various regions was seen to be statistically significant (*p* = 0.001). On pair-wise comparison, outer nasal region CT was seen to be significantly lower than central CT (*p* = 0.042) and inner nasal CT (*p* = 0.007). Other regions were not significantly different in terms of CT. Similarly, with respect to the three rings, overall within subject contrasts was statistically significant (*p* = 0.01), and on pairwise comparison, outer ring CT was significantly less than central (*p* = 0.04) and inner ring (*p* = 0.01) CT. Quadrant-wise CT values were not significantly different (within subject contrast *p*-value = 0.19) CVI values were also not significantly different between the different zones (within subject contrast *p*-value of 0.13,0.18 and 0.12 for regions, rings and quadrants, respectively).

#### 3.1.2. Fellow Eyes

Repeated measures ANOVA showed that outer nasal CT was significantly lower than inner nasal (*p* = 0.02), inner temporal (*p* = 0.02) and outer temporal CT (*p* = 0.01) on pairwise comparison (overall within subject contrast, *p* = 0.01). On the other hand, overall, within subject contrasts the *p*-value was 0.07 on analyzing the various rings, with outer ring CT being significantly lower than inner ring CT (*p* = 0.04) on pairwise comparison. In analysis of various quadrants, nasal quadrant CT was significantly lower than superior quadrant CT (*p* = 0.01) and temporal quadrant CT (*p* = 0.02) on pairwise comparison (overall within subject contrast, *p* = 0.01). CVI values were not significantly different (within subject contrast *p*-values of 0.74, 0.36 and 0.81 for regions, rings and quadrants, respectively).

#### 3.1.3. Healthy Controls

Central CVI was lower than outer temporal (*p* < 0.01), and outer inferior (*p* = 0.001); Inner nasal CVI was lower than inner superior (*p* = 0.012), inner temporal (*p* = 0.017), outer temporal (*p* < 0.001) and outer inferior (*p* = 0.002); Outer nasal CVI was lower than outer temporal (*p* < 0.001) and outer inferior (*p* = 0.029); Outer ring CVI was higher than inner ring (*p* = 0.04) and central CVI (*p* = 0.005); temporal CVI (*p* < 0.001) and inferior CVI (*p* = 0.013) were higher than central; superior CVI was higher than nasal CVI (*p* = 0.041); nasal was lower than inferior (*p* = 0.002) and temporal CVI (*p* < 0.001). None of the CT values were significantly different

### 3.2. Between Group Comparison

The CT values in the CSCR group were statistically higher than fellow eyes in central (*p* = 0.02), inner superior (*p* = 0.03), inner temporal (*p* = 0.02), inner nasal (*p* < 0.01), inner inferior (*p* = 0.04), outer temporal (*p* = 0.01), outer nasal (*p* < 0.01) and outer inferior (*p* < 0.01) regions. Similarly, the inner ring (*p* < 0.01), outer ring (*p* < 0.01); superior (*p* = 0.05), temporal (*p* = 0.01), nasal (*p* < 0.01) and inferior (*p* < 0.01) quadrants were significantly higher in the CSCR eyes. None of the CVI values were significantly different. A summary of the comparison between the two groups is given in Table 1.

**Figure 2:**
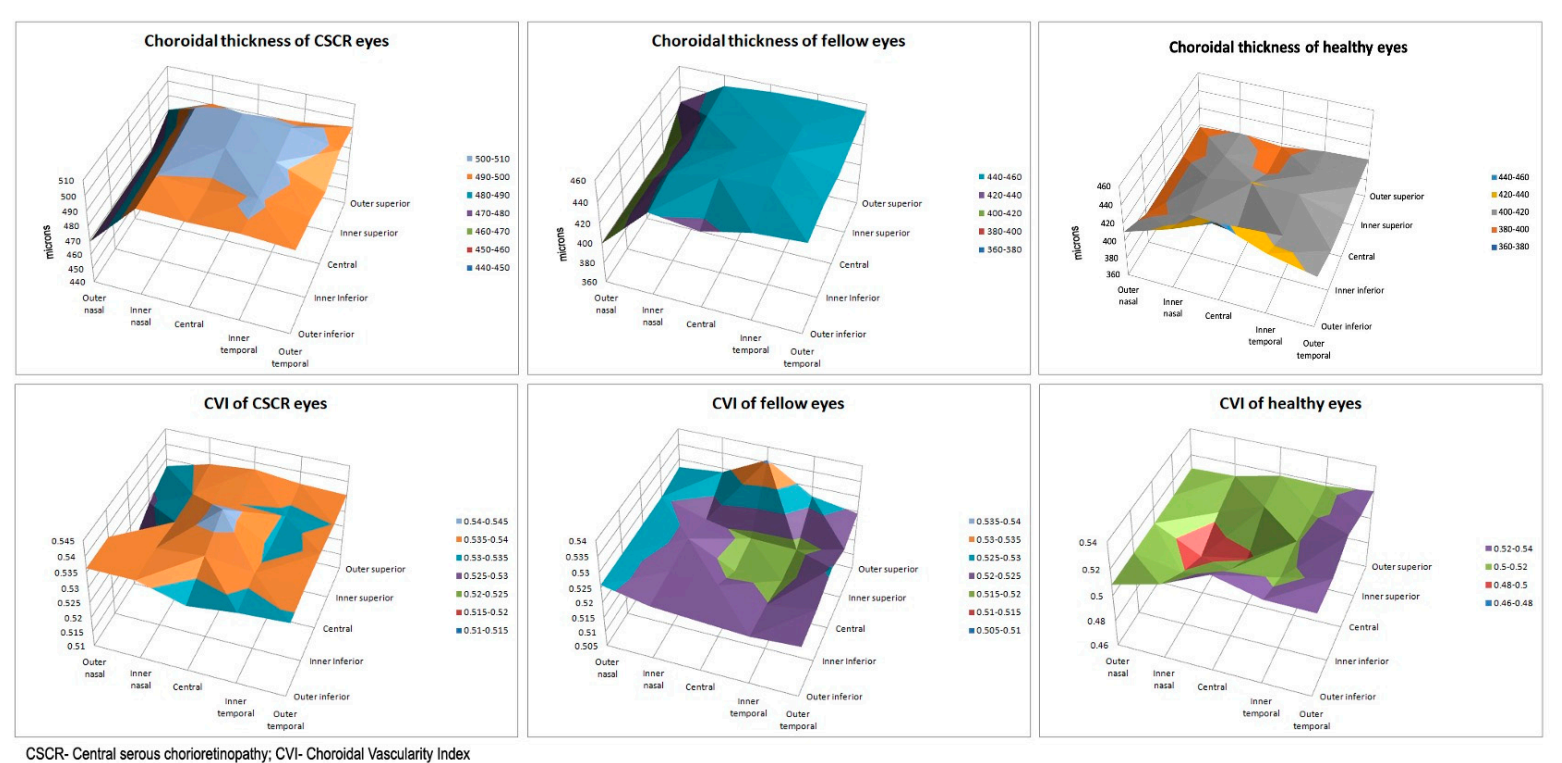
Chart showing comparison of choroidal thickness and choroidal vascularity index of CSCR and fellow eyes.

Figure 2 shows the graphical representation of the topographical distribution of CT and CVI values. A smooth rise in the CT values was seen towards the central area in CSCR and fellow eyes. A sharp rise in CVI was seen centrally and superiorly in CSCR eyes but a central reduction was seen in fellow eyes. A nasal decline in CVI values was seen in the CSCR group in contrast to an increasing trend in fellow eyes.

### 3.3. Co-Relation between CT and CVI

#### 3.3.1. Comparison of Average of Regions

**Figure 3:**
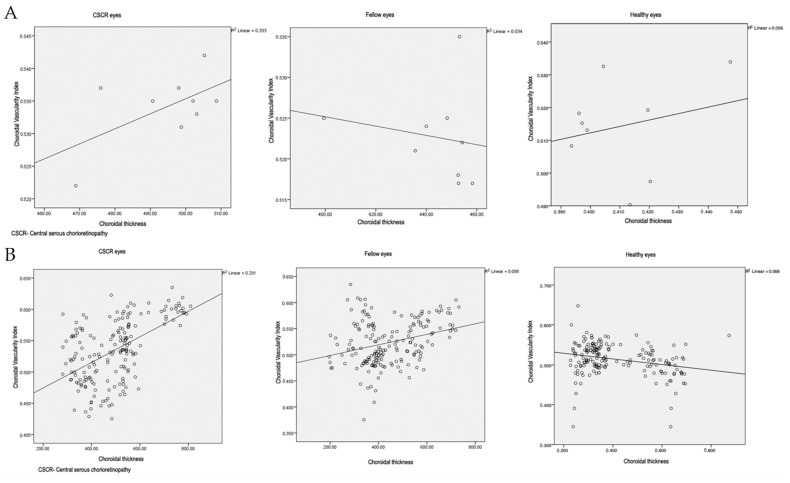
Chart showing the correlation of choroidal vascularity index and choroidal thickness values in the two groups (**A**) Region-wise and (**B**) Overall.

In CSCR eyes, a positive correlation was seen between CT and CVI values (*r* = 0.58, *p* = 0.1). There was a weak correlation between CT and CVI in fellow eyes (*r* = −0.183, *p* = 0.64) and healthy eyes (*r* = 0.236, *p* = 0.54) (Figure 3A). The correlations were not statistically significant.

#### 3.3.2. Comparison of Individual Values

On potting all the CVI values against the corresponding CT values, a positive correlation was seen in CSCR eyes (*r* = 0.54, *p* < 0.01), which was slightly weaker in fellow eyes (*r* = 0.3, *p* < 0.01) and a negative correlation was seen in healthy eyes (*r* = −0.262, *p* < 0.01) (Figure 3B).

## 4. Discussion

In this study, the choroidal thickness was found to be significantly higher in all the sub-fields except inner inferior and outer superior sub-fields in CSCR eyes compared to fellow eyes and healthy normal controls. Although the difference in CVI was not seen to be significantly different between the three groups, there was an increasing trend of CVI towards the center of macula in CSCR eyes while there was a decreasing trend towards the center of macula in fellow eyes and healthy controls.

Acute CSCR is characterized by choroidal congestion which can be seen on OCT as an increased choroidal thickness [1,2,12]. This has been described in multiple studies in the past using sub-foveal CT as an indirect marker of the overall choroidal status. However, this gives limited information about the focal changes occurring in different areas of choroid. We found that the CT values were consistently higher in CSCR eyes than the fellow eyes in multiple sub-fields. This finding was consistent with previous reports on acute CSCR [1]. We also found that the inner ring choroid was significantly thicker than the outer ring in both CSCR and their fellow eyes. This was in agreement with previous studies reporting a decreasing trend of choroidal thickness as we move to the periphery [13].

The insignificant difference in CVI values suggests a proportional increase in choroidal luminal and stromal area. However, on plotting a surface chart, it was seen that there was a rising trend towards the center of macula in CSCR eyes while there was a central dip in fellow eyes and healthy normal eyes. CVI is directly proportional to the choroidal vessel luminal area but inversely proportional to the total choroidal area [7]. As we move towards the center of macula, an increase in the choroidal thickness is seen. This leads to a proportional decrease in CVI values in normal eyes [8]. A similar phenomenon was seen in the fellow and normal eyes in our study. On the other hand, the increasing CVI towards the center of macula in CSCR eyes also suggests a disproportionate increase in the choroidal luminal area. This indicates a generalized change in choroidal vascularity in acute CSCR, with maximum effect at the sub-foveal choroid. Several theories have been put forth to explain such changes in the choroid. One unique characteristic of choroid lies in the presence of non-vascular smooth muscle cells (NVSMC) [14,15]. These specialized cells are more concentrated in the macular area and are responsive to autonomic regulation [15]. An up-regulation of sympathetic tone has been described in CSCR, which may result in the stretching of these cells [15,16,17]. This allows for enlargement of choroidal vascular caliber and expansion of the choroidal stromal area for accumulation of fluid. Furthermore, in CSCR, activation of mineralocorticoid receptors results in hyperpolarisation of choroidal endothelial cells, which causes up-regulation of Ca^2+^ -activated K+ channels [18]. This culminates in dilatation of the choroidal blood vessels. The higher CVI at the sub-foveal area in our study suggest that the choroidal vessels in this area are more responsive to the inciting stimuli compared to the rest of the choroid. In addition to this, the CVI seemed to increase superiorly in CSCR eyes. As mentioned previously, this is also accompanied by an increased choroidal thickness superiorly. While the overall choroidal thickness was seen to decrease superiorly, an increase in luminal volume was seen only in the central and superior macula (resulting in an increase in CVI). It could also partly explain the fact that the most common site of leak has been reported to be the superior half of the posterior pole in CSCR [19]. The pachychoroid spectrum of diseases also demonstrate various degrees of vortex vein anastomosis in the posterior pole that contribute to the luminal area measurement of CVI [20]. This could possibly influence the distribution of CVI seen in our study.

Another interesting observation in this study was the correlation between CVI and CT. On comparing the averages from various zones, a negative correlation was seen between CT and CVI in fellow eyes, and a weak positive correlation was seen in normal eyes. On the other hand, a positive correlation was seen in CSCR eyes. Similarly, on plotting the overall CVI data against the CT data, a weak positive correlation was seen in fellow eyes and a negative correlation was seen in healthy eyes, while a stronger positive correlation was seen in CSCR eyes. A possible explanation could be the differential response of various areas of choroid to the inciting stimuli, resulting in a non-homogenous distribution of vessel dilatation and stromal expansion in CSCR eyes.

Apart from the retrospective study design, one of the major limitations of the study was the small sample size. The smaller sample size was due to strict inclusion criteria of unilateral disease and no changes in the fellow eye. Second, choroidal vascular architecture has been seen to change depending upon the site of leak. This correlation was not taken into consideration, which could have affected the results in the CSCR group. Third, the study evaluated only the macular area, which cannot be extrapolated to the whole of the choroid.

## 5. Conclusions

In conclusion, we report CVI and choroidal thickness changes in both eyes of patients with unilateral CSCR. We noted an increasing trend in CVI towards the center of the macula and superiorly in CSCR eyes, in contrast to a decreasing trend at the central sub-fovea in fellow eyes and healthy eyes. Furthermore, the correlation between CVI and CT was altered in CSCR eyes, compared to fellow eyes and healthy eyes. This correlation could be used as a guide to detect sub-clinical activity, although future studies with a larger sample size are required to validate the association.

## Figures and Tables

**Table 1:** Comparison of CT and CVI values in CSCR and their fellow eyes.

			CSCR	Fellow	*p*-Value	Normal
CT (µm)	**Region**	**Central**	505.27 ± 124.22	452.76 ± 127.52	0.02	420.32 ± 139.47
		**Inner Superior**	508.7 ± 126.74	452.6 ± 124.82	0.03	397.15 ± 127.13
		**Inner Temporal**	503.1 ± 133.16	458.25 ± 126.88	0.02	419.53 ± 147.31
		**Inner Nasal**	502.08 ± 121.71	439.99 ± 120.51	<0.01	413.42 ± 145.16
		**Inner Inferior**	498 ± 132.73	454.21 ± 146.87	0.04	398.87 ± 145.09
		**Outer Superior**	475.94 ± 139.48	453.15 ± 136.94	0.26	396.14 ± 138.61
		**Outer Temporal**	490.66 ± 149.43	448.25 ± 129.12	0.01	404.40 ± 151.91
		**Outer Nasal**	468.89 ± 124.58	399.44 ± 115.23	<0.01	393.59 ± 158.89
		**Outer Inferior**	498.74 ± 132.62	435.64 ± 124.23	<0.01	447.50 ± 181.11
	**Ring**	**Inner ring**	502.97 ± 126.36	451.26 ± 121.97	<0.01	407.24 ± 135.23
		**Outer ring**	483.56 ± 132.51	434.12 ± 119.79	<0.01	410.41 ± 144.37
	**Quadrant**	**Superior**	492.32 ± 130.9	452.87 ± 122.76	0.05	396.64 ± 128.45
		**Temporal**	496.88 ± 140.71	453.25 ± 126.63	0.01	411.96 ± 148.86
		**Nasal**	485.49 ± 122.1	419.71 ± 115.87	<0.01	403.51 ± 151.42
		**Inferior**	498.37 ± 129.77	444.93 ± 123.99	<0.01	423.19 ± 147.97
	**Overall average**		494.6 ± 128.14	443.81 ± 120.26	0.03	410.10 ± 148.29
CVI	**Region**	**Central**	0.542 ± 0.05	0.517 ± 0.046	0.06	0.497 ± 0.019
		**Inner Superior**	0.535 ± 0.051	0.518 ± 0.046	0.18	0.515 ± 0.028
		**Inner Temporal**	0.533 ± 0.052	0.517 ± 0.042	0.20	0.516 ± 0.031
		**Inner Nasal**	0.535 ± 0.056	0.524 ± 0.042	0.41	0.490 ± 0.029
		**Inner Inferior**	0.537 ± 0.051	0.522 ± 0.05	0.24	0.513 ± 0.047
		**Outer Superior**	0.537 ± 0.046	0.535 ± 0.033	0.79	0.518 ± 0.047
		**Outer Temporal**	0.535 ± 0.051	0.525 ± 0.041	0.33	0.533 ± 0.023
		**Outer Nasal**	0.522 ± 0.045	0.525 ± 0.046	0.79	0.508 ± 0.042
		**Outer Inferior**	0.531 ± 0.041	0.521 ± 0.04	0.19	0.530 ± 0.032
	**Ring**	**Inner ring**	0.535 ± 0.045	0.52 ± 0.032	0.11	0.509 ± 0.028
		**Outer ring**	0.531 ± 0.038	0.527 ± 0.032	0.48	0.523 ± 0.037
	**Quadrant**	**Superior**	0.536 ± 0.043	0.527 ± 0.034	0.27	0.517 ± 0.042
		**Temporal**	0.534 ± 0.05	0.521 ± 0.04	0.23	0.526 ± 0.025
		**Nasal**	0.529 ± 0.047	0.525 ± 0.04	0.67	0.499 ± 0.032
		**Inferior**	0.534 ± 0.043	0.522 ± 0.037	0.14	0.522 ± 0.036
	**Overall average**		0.534 ± 0.041	0.523 ± 0.031	0.78	0.514 ± 0.035

CT—Choroidal thickness; CVI—Choroidal vascularity index; CSCR—Central serous chorioretinopathy.

## Data Availability

The data presented in this study are available on request from the corresponding author.
